# Comprehensive Analysis of Pyroptosis-Related Genes and Tumor Microenvironment Infiltration Characterization in Papillary Renal Cell Carcinoma

**DOI:** 10.3389/fmolb.2022.871602

**Published:** 2022-03-23

**Authors:** Chiyu Zhang, Ruizhen Huang, Xiaoqing Xi

**Affiliations:** Department of Urology Surgery, The Second Affiliated Hospital of Nanchang University, Nanchang, China

**Keywords:** pyroptosis, papillary renal cell carcinoma, gene mutation, prognostic model, drug sensitivity

## Abstract

**Background:** Immunotherapy has emerged as an important technique for treating a variety of cancers. The dynamic interplay between tumor cells and invading lymphocytes in the tumor microenvironment is responsible for the good response to immunotherapy (TME). Pyroptosis, or inflammation-induced cell death, is closely linked to a number of cancers. However, in papillary renal cell carcinoma (KIRP), the association between pyroptosis and clinical prognosis, immune cell infiltration, and immunotherapy impact remains unknown.

**Methods:** We carefully investigated the link between pyroptosis and tumor growth, prognosis, and immune cell infiltration by evaluating 52 pyroptosis-related genes. The PRG score was utilized to measure a single tumor patient’s pyroptosis pattern. After that, we looked at how well these values predicted prognoses and therapy responses in KIRP.

**Results:** We discovered that PRG differences between subgroups were linked to clinical and pathological aspects, prognosis, and TME in two separate genetic subtypes. After that, a PRG score for estimating overall survival (OS) was developed, and its predictive potential in KIRP patients was confirmed. As a result, we developed a very precise nomogram to improve the PRG score’s clinical usefulness. A low PRG score, which is determined by mutation load and immune activation, suggests a good chance of survival. Furthermore, the PRG score was linked to chemotherapeutic drug sensitivity in a substantial way.

**Conclusions:** The possible functions of PRGs in the TME, clinical and pathological characteristics, and prognosis were established in our thorough investigation of PRGs in KIRP. These results might help us better understand PRGs in KIRP and offer a new avenue for prognostic evaluation and the development of more effective immunotherapy treatments.

## Background

Renal cell carcinoma (RCC) is a broad term that refers to a variety of malignancies. Each kind has different histologic characteristics, a distinct genetic profile, and a varied clinical history and response to treatment (Moch et al., 2016). Papillary renal cell carcinoma (KIRP) is the second most frequent RCC subtype, accounting for 10–15% of patients (Akhtar et al., 2019). Papillary renal cell carcinoma is divided into two categories based on their histological characteristics (Mendhiratta et al., 2021). When compared to non-papillary RCC subtypes, KIRP often present as homogenous, solid masses with relative hypovascularity (Vikram et al., 2009; Tordjman et al., 2020). Targeted therapy for KIRP has previously failed due to a lack of understanding of the molecular underpinnings of these cancers (Williamson, 2021). It is hoped that, as we get a better understanding of the etiology of the pathogenesis of KIRP, effective targeted therapeutics will emerge in due course.

Pyroptosis, defined as a programmed cell death caused by the Gasdermin family, is accompanied by inflammatory and immunological responses (Xia et al., 2019; Meng et al., 2021; Wu et al., 2021). Pyroptosis and cancer have a complicated interaction; pyroptosis can prevent tumor incidence and progression while simultaneously acting as a factor promoting inflammation to generate a milieu conducive to tumorigenesis (Wu et al., 2021; Xiang et al., 2021). The fundamental cause of pyroptosis is the stimulation of inflammasome, which occurs through the caspase inflammasome pathways (Zheng and Li, 2020; Song et al., 2021). The importance of pyroptosis in the TME is becoming clearer, while the molecular basis of pyroptosis in the KIRP immune microenvironment is yet unknown.

Employing computational techniques, this work analyzed the mRNA transcription of pyroptosis-related genes (PRGs) in detail and created a complete picture of the tumoral immune landscape. To begin with, patients with KIRP were divided into two distinct subgroups based on their PRG transcription levels. On the basis of differentially expressed genes (DEGs), patients were then divided into three gene subgroups. Additionally, we developed a grading scale to estimate overall survival (OS) and to define the immunological landscape of KIRP, which effectively predicted clinical outcomes and immunotherapy responsiveness.

## Materials and Methods

### KIRP Data Source and Preprocessing

The TCGA platform was used to obtain the transcriptome RNA sequences and clinical data for 471 KIRP samples. The Gene Expression Omnibus (GEO; https://www.ncbi.nlm.nih.gov/geo/) platform was used to collect the GEO cohort (GSE2748) KIRP samples. Incomplete clinical information was deleted from samples, and FPKM values in TCGA-KIRP were transformed to Transcripts Per Kilobase Million (TPM) values and utilized for copy number variation (CNV) analysis (Zhao et al., 2021). The transcriptome RNA sequences from the TCGA-KIRP and GSE2748 datasets were combined after correction. R software version 4.1.1 was used to process the raw data.

### Unsupervised Clustering Analysis of PRGs

First and foremost, PRGs were identified from published literature (Man and Kanneganti, 2015; Wang and Yin, 2017; Karki and Kanneganti, 2019). The transcription of 52 PRGs was utilized to identify distinct pyroptosis types and to identify patients for further research using an unsupervised clustering approach. To do the aforementioned study, we utilized the R package “ConsensuClusterPlus” and 1,000 repeats to ensure clustering stability (Wilkerson and Hayes, 2010). Our best guess for how many clusters there should be was arrived at by using the consensus clustering method. Gene set variation analysis (GSVA) was performed on the MSigDB hallmark gene set in order to investigate the variations in PRGs that occur throughout cellular mechanisms. Furthermore, the Kaplan–Meier curves generated by the R packages “survival” and “survminer” were used to investigate the differences in overall survival across subtypes.

### Relationship Between Molecular Subgroups and TME in KIRP

We utilized the ESTIMATE method to assess each patient’s immunological and stromal scores. KIRP samples’ fractions of 22 different kinds of human immune cells were also determined using the CIBERSORT technique (Chen et al., 2018). To evaluate the degree of immune cell infiltration in the TME of KIRP, a single-sample gene set enrichment analysis (ssGSEA) approach was utilized in conjunction with the other techniques (Subramanian et al., 2005).

### Identification of DEGs in Various PRG Clusters

We utilized the R package “limma” to compare DEGs between PRG clusters (Ritchie et al., 2015). The Gene Ontology (GO) enrichment and Kyoto Encyclopedia of Genes and Genomes (KEGG) pathway analyses were carried out using the “clusterProfiler” program (Gene Ontology Consortium, 2015; Kanehisa et al., 2017).

### Generation of PRG Score

To objectively measure pyroptosis in individual KIRP patients, a score system was developed. The following is the procedure for developing the scoring system: The DEGs found in various pyroptosis clusters were first standardized across all samples, and then the overlap genes were retrieved. Then, for each gene, we ran a univariate Cox regression analysis. For the following phase of the investigation, a list of genes with a high prognosis was created. The following formula was used to determine pyroptosis scores using principal component analysis (PCA):

PRG Score = Σ (Expi * Coefi)

### The Creation and Testing of a Nomogram Grading System

The clinical characteristics and risk score from the independent prognostic study were used to generate a nomogram using the “rms” program (Park, 2018). In the nomogram scoring approach, a score was given to each variable, and the total score was derived by adding the scores from all variables in each sample. With the help of nomogram calibration plots, it was possible to see the relationship between surviving events and their virtually observed consequences.

### Analyzing Drug Susceptibility and Mutations

The mutation annotation file from the TCGA was developed using the “maftools” R package to determine the genetic alterations of KIRP patients in various categories (Mayakonda et al., 2018). For each patient with KIRP in the two groups, we estimated the tumor mutation burden (TMB) value. Using the “pRRophetic” software, we estimated the semi-inhibitory concentration (IC50) values of medications frequently used to treat KIRP in patients to see if there were any variations in their therapeutic effects (Geeleher et al., 2014).

## Results

### Genetic and Transcriptional Variation of PRGs in KIRP

**FIGURE 1:**
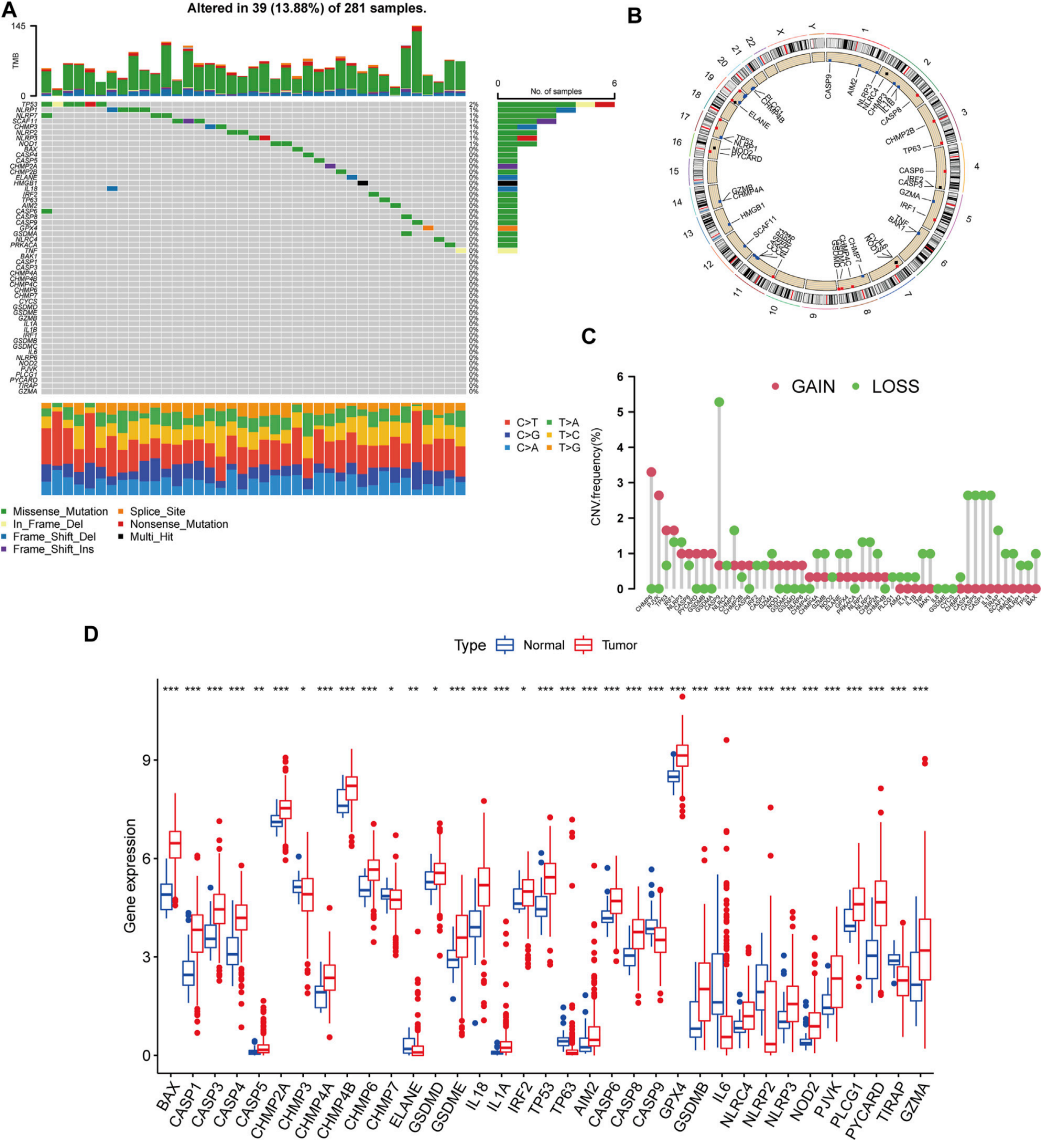
Genetic and transcriptional variation of PRGs in KIRP. **(A)** The number of genetic mutations found in KIRP patients. The PRGs’ mutation frequency was indicated by the number on the right. Each column represents one of the 39 TCGA samples in which the PRGs had at least one genetic change. **(B)** On the chromosomes, the position of the PRGs CNV changes. **(C)** The PRGs’ CNV mutation frequency. The frequency of modification was displayed in the column. The frequency of amplification is shown by a red dot, whereas the frequency of deletion is indicated by a green dot. **(D)** PRGs differential expression study in normal and KIRP tissues. *p < 0.05; **p < 0.01; ***p < 0.001.

We began by looking at their CNV and mutation frequency in TCGA-KIRP. PRG mutations were found in 39 of 281 TCGA-KIRP samples, accounting for 13.88 percent of the total. The top four PRGs with the greatest mutation frequencies were TP53 (2%), NLRP1 (1%), NLRP7 (1%), and SCAF11 (1%) (Figure 1A). Further investigating the predictive impact of PRGs, we discovered that most PRGs, such as TP53, GZMB, AIM2, CHMP7, and GSDMC, were substantially linked with the overall prognosis of KIRP patients. Figure 1B depicts the 39 PRG positions on the chromosomal CNV alterations. CNV change was widespread in the 52 PRGs that exhibited mostly copy number amplification, such as CHMP6 and PJVK, whereas CASP9, CASP4, CASP5, CASP1, and IL18 showed considerable copy number decrease (Figure 1C). The levels of PRGs in KIRP patients and renal tissues were examined to see whether the genetic variants mentioned above had an influence on PRG transcription in KIRP patients (Figure 1D). According to our results, variations in CNV may be major factors driving changes in PRG regulator expression. CNV-amplified PRG transcription was considerably greater in KIRP compared to normal renal tissue (e. g., CHMP6 and PJVK) and vice versa (e. g., CHMP3, CHMP7, ELANE, CASP9, NLRP2, and TIRAP). This study found considerable PRG inherited heterogeneity and variance in KIRP patients, indicating that PRG transcription imbalances were important in the onset and development of KIRP.

### Construction of Pyroptosis Subtypes in KIRP

**FIGURE 2:**
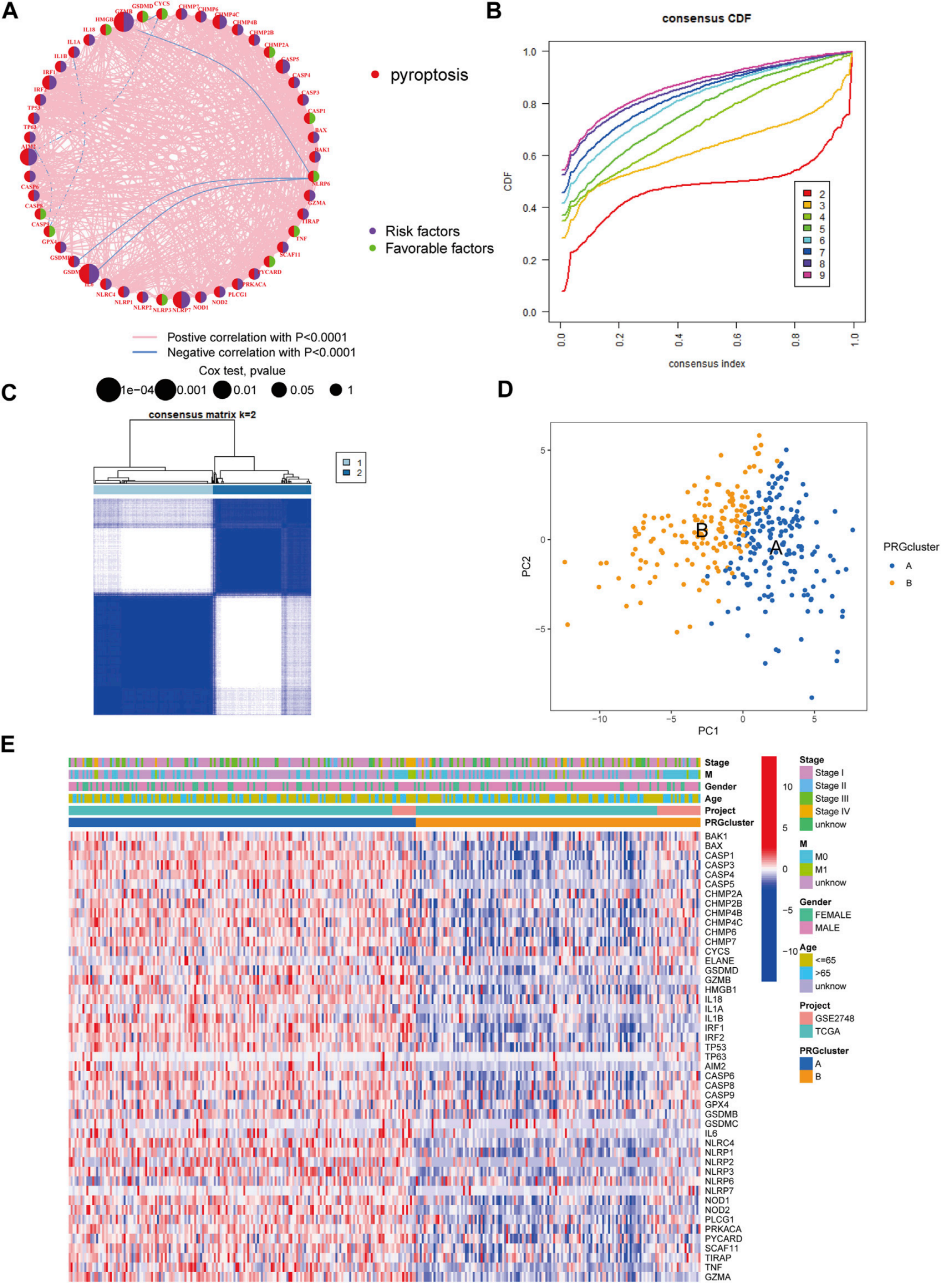
Unsupervised cluster analysis divided KIRP patients into subclusters. **(A)** Interactions of PRGs in KIRP. **(B)** The cumulative distribution function (CDF) for consensus clustering. **(C)** Heatmap of the consensus matrix identifying two clusters and their correlation area. **(D)** Principal component analysis (PCA) between the two subtypes. **(E)** PRG heatmap in KIRP between two distinct subtypes.

The patients from two suitable KIRP cohorts (TCGA-KIRP and GSE2748) were included in our study for additional analysis in order to thoroughly recognize the PRG expression levels implicated in carcinogenesis. We created a pyroptosis network map to demonstrate the interactions of PRGs in KIRP patients and their relationship to prognosis in these patients (Figure 2A). We employed a consensus clustering technique to identify patients according to the transcript level of the 52 PRGs to learn more about their expression features in KIRP. Our findings indicated that k = 2 was the best choice for categorizing the whole patients into subgroup A (n = 177) and subgroup B (n = 146) (Figures 2B,C). According to the results of the PCA analysis, the PRG expression patterns of the two subgroups were considerably different (Figure 2D). In addition, comparing the clinicopathological characteristics of various KIRP subtypes revealed substantial disparities in PRG transcription and clinical features (Figure 2E). Cluster A had the highest level of expression for PRGs.

**FIGURE 3:**
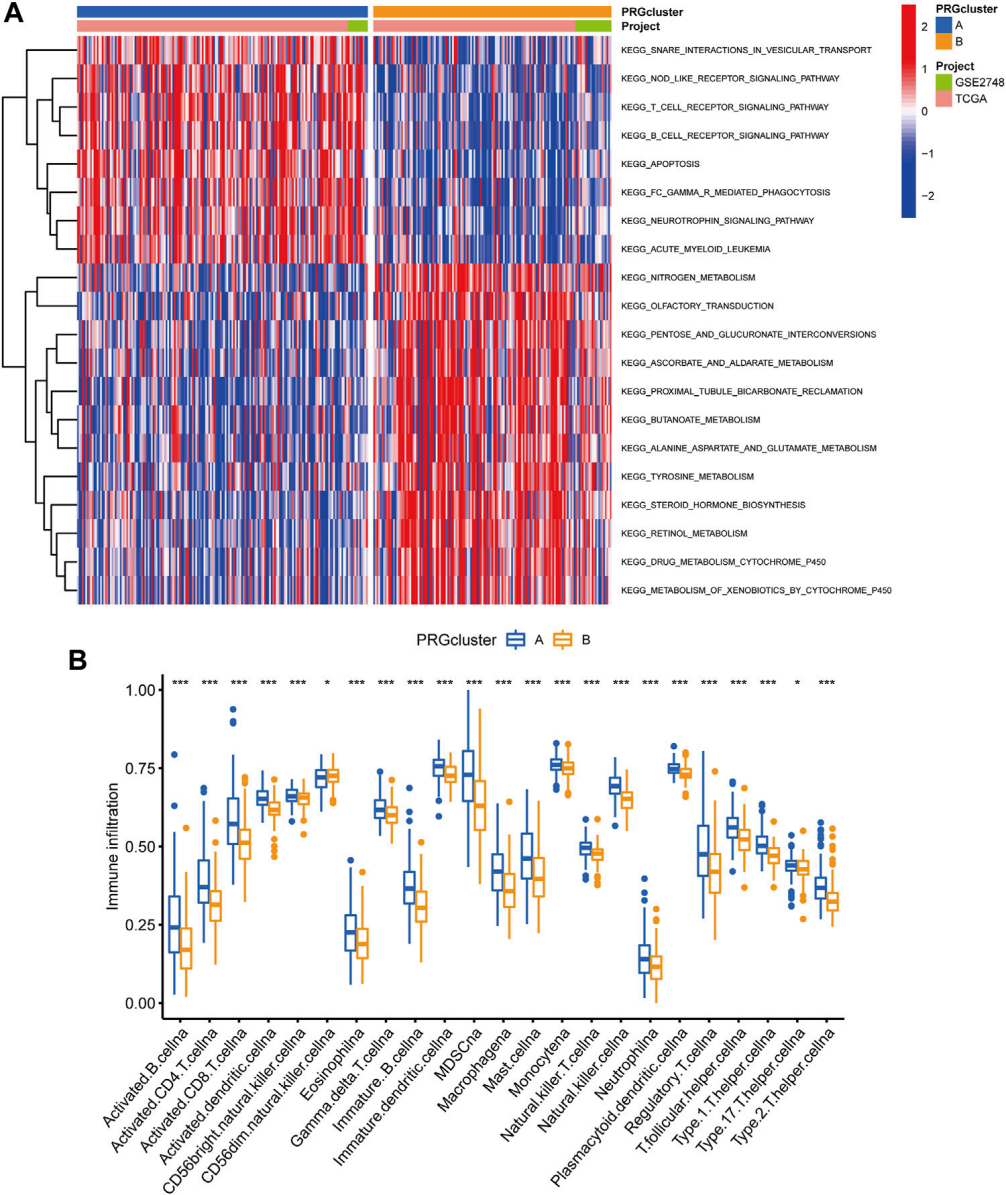
Analysis of GSVA enrichment and TME immune cell infiltration. **(A)** The functional pathways of two PRG subgroups were identified using GSVA enrichment. **(B)** Immune cell infiltration levels differ between PRG subgroups.

Following that, we used GSVA enrichment to investigate the biologic effects of several PRG groups. There was significant enrichment of immune cell activation pathways in PRG cluster A, including T cell and B cell receptor signaling pathways (Figure 3A). Then, we employed ssGSEA to compare immune cell infiltration in the tumor microenvironment amongst distinct PRG clusters. Surprisingly, we found a considerable variation in immune cell concentration amongst PRG clusters, with cluster A was considerably enriched in immune cells (Figure 3B).

### PRG Signature Generation

**FIGURE 4:**
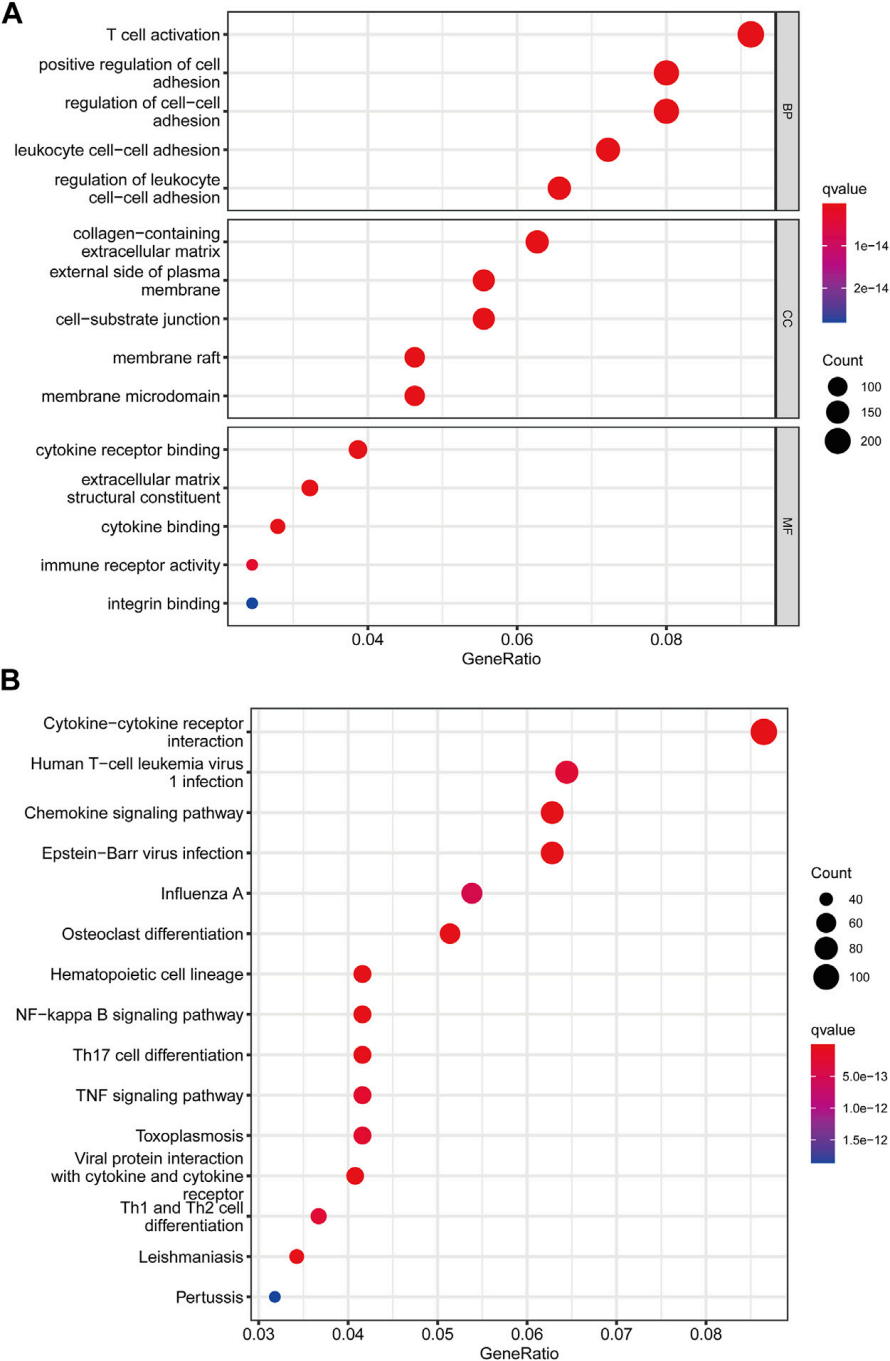
Functional enrichment analysis among two pyroptosis subtypes. **(A)** Enrichment of GO functions. **(B)** The KEGG pathway.

We utilized the “limma” R package to explore 2461 PRG phenotype-associated DEGs, which were designated as PRG signature genes, to learn more about the probable biologic activities of distinct PRG clusters. Surprisingly, it was shown that DEGs are greatly overrepresented in immune-related pathways. DEGs were found to be concentrated in biological processes of BP related to T cell activation and leukocyte cell-cell adhesion. DEGs were considerably enriched in cytokine receptor binding, cytokine binding, and immune receptor activity throughout MF processes (Figure 4A). Additionally, DEGs were considerably enriched in immune-related pathways in KEGG pathway enrichment analyses: cytokine-cytokine receptor interaction, Human T-cell leukemia virus infection, and chemokine signaling pathway (Figure 4B).

**FIGURE 5:**
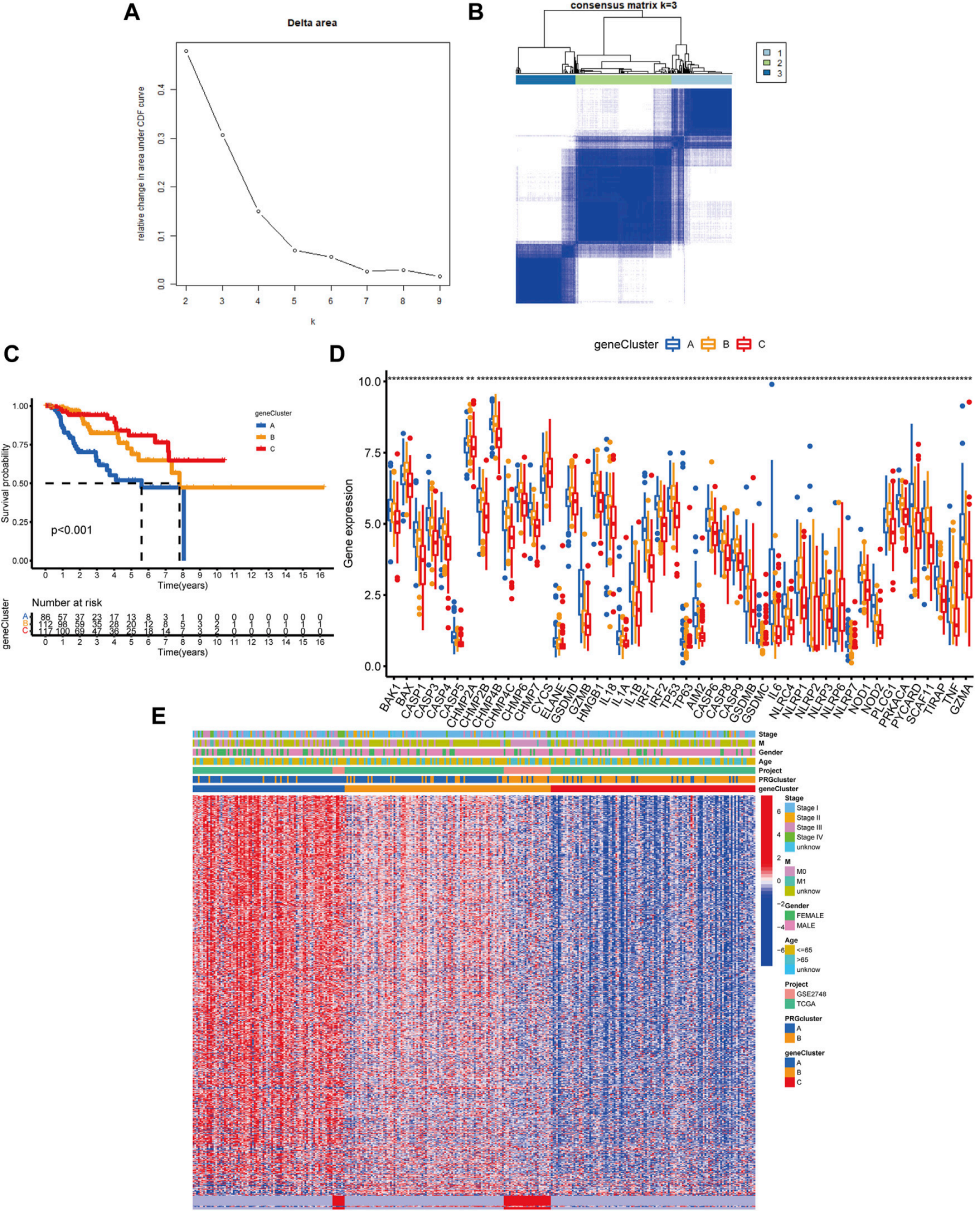
Classification of genetic subgroups. **(A)** Changes in the area under the CDF curve. **(B)** The K = 3 consensus clustering matrix. **(C)** Survival curves for subgroups A, B, and C. **(D)** PRG expression between gene clusters. **(E)** Heatmap of genes based on unsupervised clustering techniques.

Following that, we used univariate Cox analysis to screen for prognostically related DEGs, yielding 659 genes related to survival time. We employed unsupervised cluster analysis to separate the KIRP patients into three gene groups based on the genes that were chosen for inclusion (Figures 5A,B). The survival research showed that patients in subgroup C had the highest overall survival rate, while those in subgroup A had the lowest OS (Figure 5C). In gene cluster A, all PRGs were found to be overexpressed (Figure 5D). This revealed that greater PRG expression might be related to a worse prognosis in KIRP patients. Furthermore, the heatmap revealed that these prognostically associated DEGs were common in subgroup A, which confirmed the findings of the previous study (Figure 5E).

### Generation of PRG the Signatures Scoring System

**FIGURE 6:**
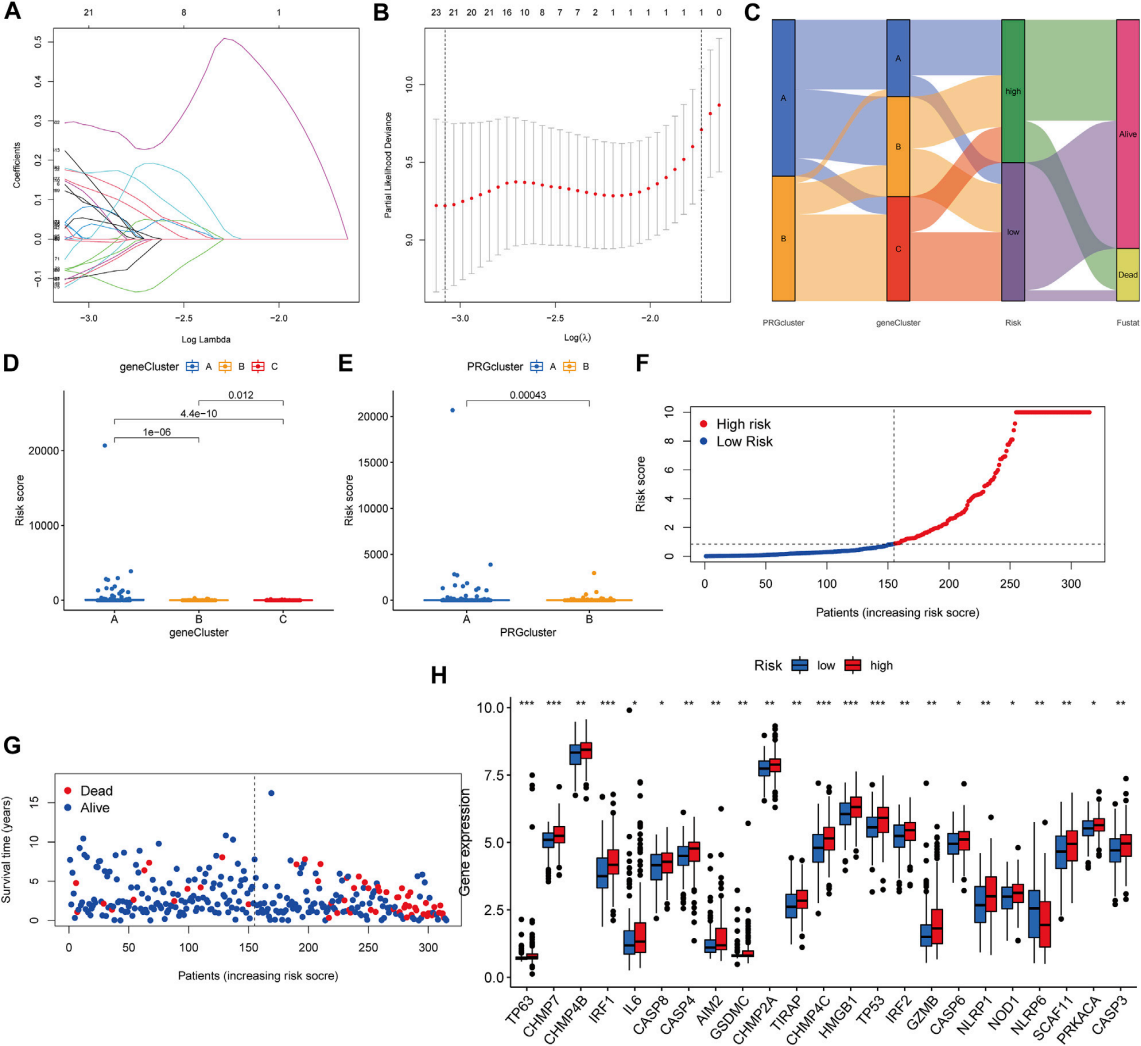
Generation of the PRG signature scoring system. **(A–B)** LASSO regression analysis and partial likelihood deviance on prognostic genes. **(C)** An alluvial plot illustrating the link between the pyroptosis cluster, the gene cluster, and the pyroptosis score. **(D)** The PRG score in different genecluster subgroups. **(E)** The distribution of PRG scores in various PRGcluster subgroups. **(F–G)** The ranked dot plot depicts the distribution of PRG scores, whereas the scatter plot depicts the survival state. **(H)** Variations in PRG expression across risk categories.

We established the PRG score, a scoring method that evaluates the pyroptosis types of each KIRP patient, to further examine the activities of pyroptosis in KIRP. In order to begin, we separate the patients into two groups: those who will be trained and those who will be tested. LASSO and multivariate Cox analyses were performed on prognostic DEGs associated with pyroptosis subtypes to further select the best prognostic features (Figures 6A,B). In order to depict variations in the characteristics of specific patients, an alluvial diagram was employed (Figure 6C). Between subgroups, we identified a statistically significant variance in PRG score. Additionally, both gene cluster A (Figure 6D) and PRG cluster A had a high PRG score (Figure 6E). On the basis of the median PRG score, patients were split into high and low-risk categories. When the PRG score is increased, the survival duration reduces, and the recurrence rate rises, as shown by the risk distribution chart for the PRG score (Figures 6F,G). Furthermore, we discovered substantial disparities in the expression of PRGs between risk categories (Figure 6H).

### Survival Prediction and Nomogram

**FIGURE 7:**
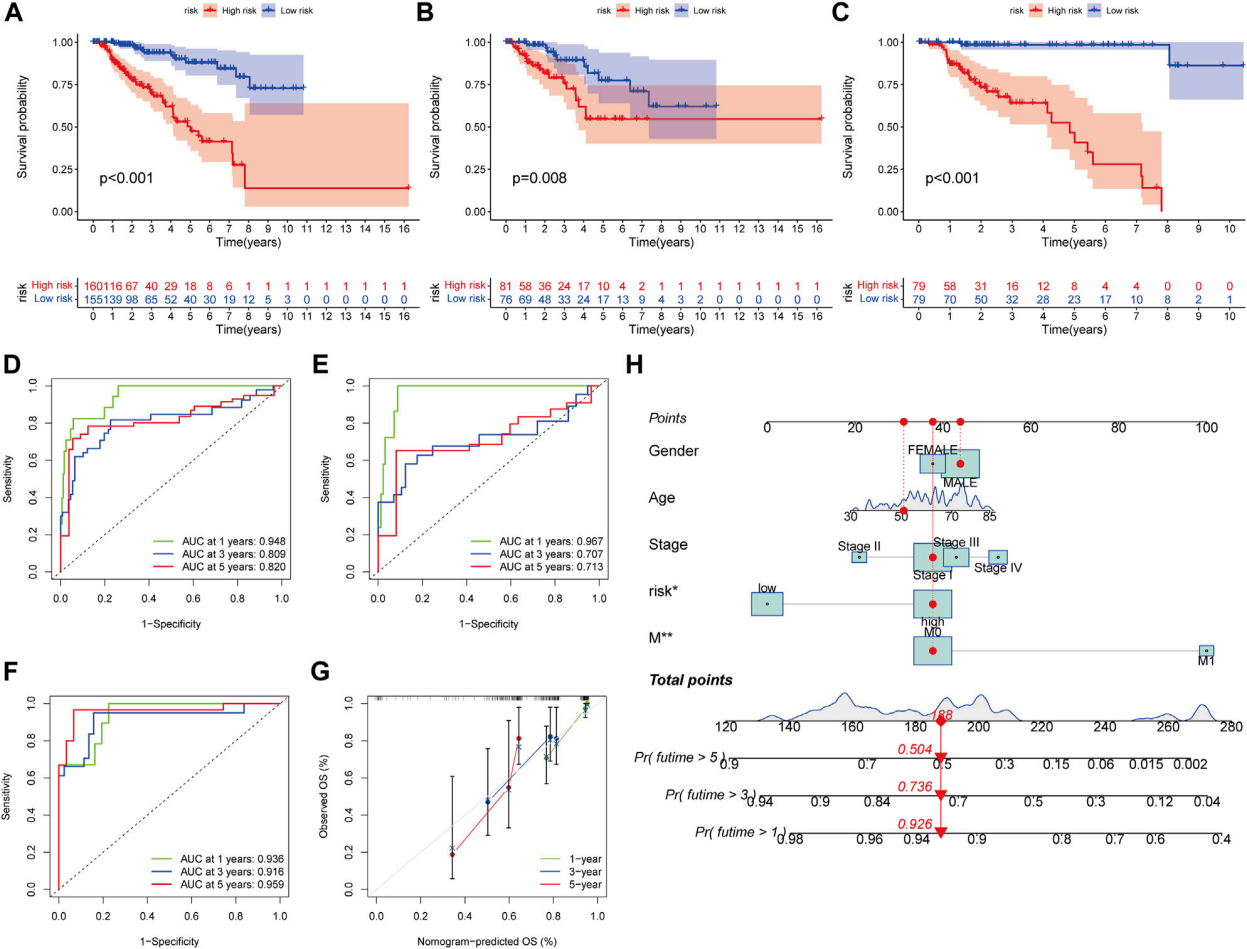
Survival prediction and nomogram. **(A–C)** Kaplan-Meier analysis of the OS between the groups in the overall, training, testing, sets. **(D–F)** ROC curves to estimate the sensitivity and specificity of 1-, 3-, and 5-year survival based on the PRG score in the overall, training, testing sets. **(G)** Calibration plots for nomograms to predict OS at 1-, 3- and 5-year. **(H)** Nomogram for predicting the 1-, 3-, and 5-year OS of KIRP patients.

In all sets (*p* < 0.001, Figure 7A), training sets (*p* = 0.008, Figure 7B), and testing sets (*p* < 0.001, Figure 7C), those with low scores had considerably better OS than subjects with elevated scores, according to the K–M curves. In all sets, AUC values of 0.948, 0.809, and 0.820 were used to reflect the 1-, 3-, and 5-year survival rates of the PRG score (Figure 7D). Similarly, the AUC in the training set was 0.967, 0.707, and 0.713 (Figure 7E), and in the test set, they were 0.936, 0.916, and 0.959 (Figure 7F). According to this, the PRG score may accurately predict the clinical outcome with KIRP patients. Because the PRG score’s clinical utility in evaluating OS in patients of KIRP is unsatisfactory, a nomogram integrating the PRG score and clinical characteristics was developed to predict survival rates (Figure 7H). The calibration chart revealed that the PRG score performed well, with a good match between the projected and actual survival rates (Figure 7G).

### Assessment of TME Between the Two Groups

**FIGURE 8:**
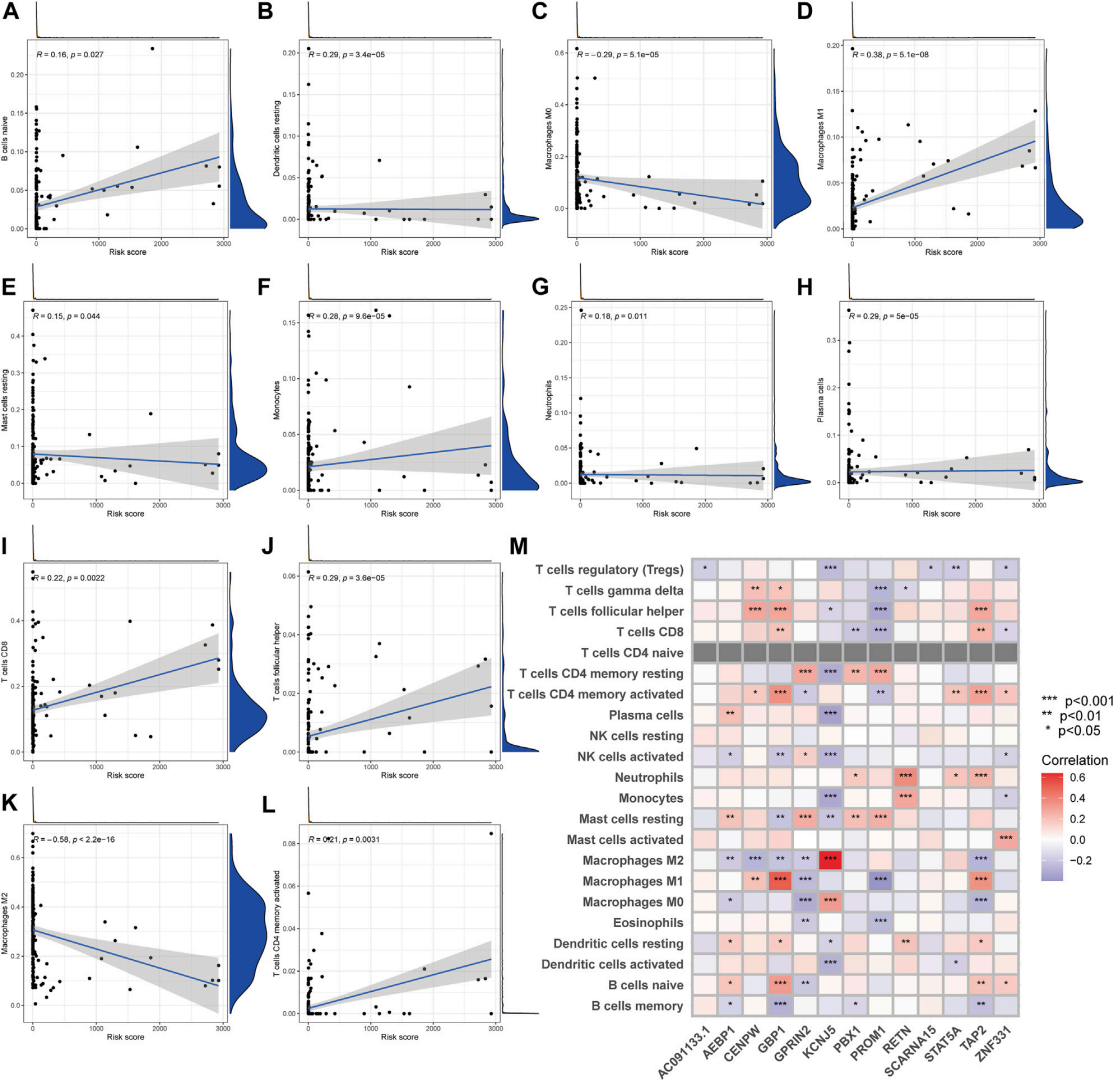
TME comparison between the two subgroups. **(A–L)** The relationship between PRG score and immune cell type. **(M)** The relationship between the abundance of 22 immune cells and the abundance of 13 genes.

The CIBERSORT technique was utilized to determine the association between the PRG score and immune cell abundance. The PRG score was favorably connected with B cells and dendritic cells resting, macrophages M1, and other cells, as indicated in the scatter diagrams, and negatively correlated with macrophages M0, macrophages M2 (Figure 8A–L). Additionally, we examined the relationship between the genes and the number of immune cells in the body. We discovered that the 13 genes were highly linked with the majority of immune cells, especially GBP1 and macrophages M1, KCNJ5 and macrophages M2 (Figure 8M).

### Analyses of Gene Mutation and Drug Sensitivity

**FIGURE 9:**
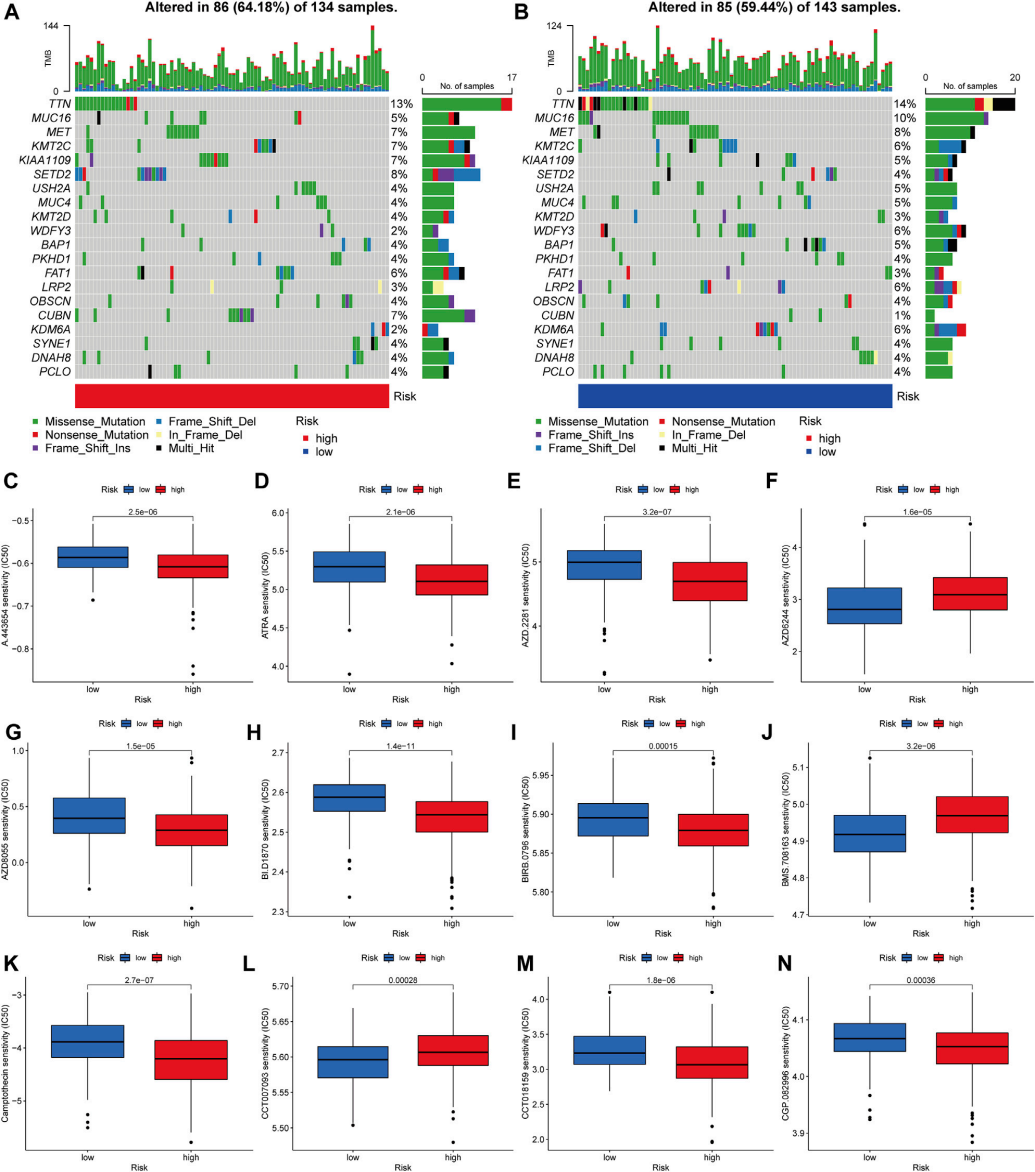
Analyses of gene mutation and drug sensitivity. **(A–B)** The waterfall plot of gene mutation characteristics generated with different scores. **(C–N)** Correlations between PRG score and drug sensitivity.

Then, in the TCGA-KIRP cohort, we looked at how the distribution of somatic mutations differed across two PRG score groups. As it turned out, the mutation rates between the two groups varied significantly (64.18 percent and 59.44 percent). TTN, MUC16, MET, KMT2C, KIAA1109, SETD2, USH2A, MUC4, KMT2D, and WDFY3 were the remarkable mutant genes in the various risk groups (Figures 9A,B). TTN, MUC16, and MET mutations were more common in patients with lower PRG scores than in those with higher PRG values. KMT2C and KIAA1109, on the other hand, have the exact opposite mutation levels. KMT2C and KIAA1109 exhibited a greater frequency of missense mutations and a lower rate of frame-shift mutations in patients with high PRG score. We next chose chemotherapeutic medications that are presently used to treat KIRP to assess the sensitivities of individuals in distinct risk categories to these treatments. We discovered individuals with high PRG scores had lower IC50 values with A-443654 (specific inhibitor of Akt), ATRA (all-trans retinoic acid), AZD-2281 (olaparib), AZD8055 (mTOR inhibitor), and BI-D1870 (RSK inhibitor). However, the IC50 values of chemotherapeutics like AZD6244 (MEK inhibitor selumetinib) and BMS-708163 (gamma secretase inhibitor) were much lower in individuals with a lower PRG score. The findings suggested that PRGs were linked to medication sensitivity (Figure 9C–N). While most of the ideas about how to diagnose and treat cancer have come from research on clear cell RCC, the unique characteristics of KIRP may have significance for illnesses.

## Discussion

KIRP is a heterogeneous subtype of RCC that differs from clear cell RCC in terms of clinicopathologic and molecular characteristics (Chen et al., 2019). When compared to clear cell RCC, KIRP is less likely to develop distant metastases upon diagnosis (Murugan et al., 2021). Because the majority of current knowledge about how to diagnose and treat renal tumors is derived from studies on clear cell RCC, the unique characteristics of KIRP may be critical to the illness (Mendhiratta et al., 2021; Yamamoto et al., 2021). For advanced KIRP, there is currently no agreed-upon treatment regimen, which poses a huge challenge for clinicians (Courthod et al., 2015). Molecularly targeted therapy and immunotherapy are also being tested in this subtype, and the results so far are favorable (Tachibana et al., 2021). KIRP has a better prognosis in the early stages than clear cell malignancies, but the situation changes drastically after the cancer has spread to distant sites (Zhao and Eyzaguirre, 2019). Pyroptosis has been linked to the emergence and progression of cancers in a growing body of data (Liu et al., 2021; Zhang et al., 2021; Zhou et al., 2021). Pyroptosis has been found to slow the development of tumors in multiple cancers (Sharma et al., 2019; Chen et al., 2020; Fenini et al., 2020). Pyroptosis can activate the immune system, inhibit tumor cell proliferation by altering the TME, and even kill malignant cells (Fu and Song, 2021; Sun et al., 2021; Wei et al., 2021). We don’t know how it affects the KIRP microenvironment or immune function just yet.

Using the TCGA–KIRP and GSE2748 cohorts, we first investigated the genetic variants and expression of PRGs. However, even while PRGs had a low mutation rate, the majority of KIRP patients’ genes were dysregulated and were linked with a poor prognosis. Second, based on 52 PRGs, we found two unique molecular subtypes. The TME’s properties changed greatly across the two subtypes. The KIRP subtypes were also distinguished by a high level of immunological activation, which included the T cell and the B cell receptor signaling pathway. Differences in mRNA transcriptome across pyroptosis subtypes were also shown to be strongly connected to PRGs and immune-associated molecular mechanisms. Third, we employed unsupervised cluster analysis to separate the KIRP patients into three gene groups based on the genes that were chosen for inclusion. Therefore, our results imply that PRGs may be useful in predicting the clinical prognosis and therapeutic responsiveness of KIRP patients. Fourth, we developed a reliable and accurate predicted score and established the predictive power. Transcript levels of the score-containing genes were also evaluated in KIRP patients. On the basis of the median PRG score, patients were split into high and low-risk categories. When the PRG score is increased, the survival duration reduces and the recurrence rate rises. Finally, by combining the score and clinical features, we created a quantitative nomogram that increased performance and made the PRG score easier to use. The prognostic model may be used to stratify the prognosis of patients, which will aid in better identifying the pathogenesis of KIRP and will bring innovation for targeted therapeutics. KIRP patients may benefit from the development of a prognostic model that can be used to stratify their prognosis. It will also assist in the better recognition of the pathogenesis of KIRP and the development of novel targeted therapeutics.

Despite recent breakthroughs in immunotherapy, KIRP patients’ outcomes remain heterogeneous, underlining the importance of TME in carcinogenesis and therapy. The cells of the TME participate in a variety of immune activations, including the pro-survival inflammatory response organized by malignancies (Seager et al., 2017). Additionally, evidence demonstrates that the TME has a major influence on tumor growth, progression, and treatment resistance (Hinshaw and Shevde, 2019). In the current research, the subtype B defined by immunological inhibition was shown to be linked with a greater PRG score, while the subtype A marked by immune activation was found to be associated with a decreased score. The features of the tumor microenvironment, as well as the abundance of various tumor-infiltrating immune cells, were found to vary considerably across the two molecular subtypes and distinct PRG scores. According to the findings of this research, PRGs play a significant role in the development of KIRP. It has been shown that subtype A and a lower PRG score are associated with greater infiltration of different activated T lymphocytes, suggesting that they play a beneficial role in the formation of KIRP. The presence of regulatory cells, which limit the immune response to tumors, has been linked with a bad prognosis (Tanaka and Sakaguchi, 2017). This is consistent with our findings that patients with subgroup B and elevated scores had more Tregs in the TME than those in the low-risk category.

Advanced KIRP treatment options have progressed significantly during the 1980s and 1990s, from cytokine and cytotoxic chemotherapy to genetic targeted treatments and immune checkpoint inhibitors at present (Ronnen et al., 2006). Despite biological differences, the early use of VEGF and mTOR inhibitors in KIRP was primarily supported by data from medical trials examining their effectiveness in clear cell RCC patients. Over the last several years, over a dozen studies have been conducted to specifically target KIRP, either on its own or in comprehensive experiments incorporating different renal tumors. Encouragingly, a series of cohort studies have revealed that these treatments are efficacious in treating KIRP (Motzer et al., 2002; Gore et al., 2009). In an attempt to elucidate the molecular basis of KIRP in the population, a comprehensive genomic analysis was performed on patients with advanced KIRP (D’Avella et al., 2020). The most frequently found mutations in individuals with type 1 illness were MET (33%), CKDN2A/B (18%), and EGFR (18%). CDKN2A/B (18%) and MET (18%) mutations were the most prevalent type 2 mutations. This work emphasizes the role of MET mutations in KIRP and another possible CDKN2A mutation for further investigation. CDK4/6 inhibitors are now licensed for the treatment of metastatic breast cancer and may be used in KIRP in the future. Whereas the best order of systemic medications is uncertain, limited therapeutic evidence shows that first-line VEGF therapy is associated with superior results when compared to mTOR inhibitors. Additionally, the ESPN study found no difference in survival time between everolimus and sunitinib as first-line therapy (Tannir et al., 2016). Due to the poor effectiveness of currently available therapies for hereditary papillary renal cell carcinoma, it is critical to understand how MET inhibitors affect it. Cabozantinib (Choueiri et al., 2016; Prisciandaro et al., 2019) and crizotinib (Schöffski et al., 2017), both of which are c-MET tyrosine kinase inhibitors, are two further targeted treatments with potential effectiveness. The objective response rate was 25.4 percent in a sample of 118 KIRP with an 11-month median follow-up, and the median duration of response was not attained, indicating potential anticancer activity in a tumor that had previously been deemed resistant to immunotherapy (McDermott et al., 2021). Combining immunotherapy with specific medicines like MET inhibitors is now being tested in clinical studies. Only p53 deletion, which has been substantially linked with poor survival, has demonstrated an association with clinical outcomes at the molecular level (Leroy et al., 2002; Perret et al., 2008; Ricketts et al., 2018). Mutations in the TP53 gene, CDKN2A gene, PBRM1 gene, and the hypermethylation genomic cluster are related to survival in KIRP (Pitra et al., 2019).

## Conclusion

Our thorough examination of PRGs revealed a complex regulatory system via which they influence TME, clinical and pathological characteristics, and prognosis. Apart from this, we also looked at how PRGs may help with immunotherapy as well as other forms of targeted therapy. The findings underscore the essential therapeutic implications of PRGs and provide novel strategies for targeting immunotherapy therapies for KIRP patients.

## Data Availability

The original contributions presented in the study are included in the article/Supplementary Material, further inquiries can be directed to the corresponding author.
